# Role of H- and D- MATE-Type Transporters from Multidrug Resistant Clinical Isolates of *Vibrio fluvialis* in Conferring Fluoroquinolone Resistance

**DOI:** 10.1371/journal.pone.0035752

**Published:** 2012-04-23

**Authors:** Priyabrata Mohanty, Arati Patel, Ashima Kushwaha Bhardwaj

**Affiliations:** Department of Human Health and Diseases, Indian Institute of Advanced Research, Gandhinagar, Gujarat, India; Indian Institute of Science, India

## Abstract

**Background:**

The study seeks to understand the role of efflux pumps in multidrug resistance displayed by the clinical isolates of *Vibrio fluvialis*, a pathogen known to cause cholera-like diarrhoea.

**Methodology:**

Two putative MATE family efflux pumps (H- and D-type) were PCR amplified from clinical isolates of *V. fluvialis* obtained from Kolkata, India, in 2006 and sequenced. Bioinformatic analysis of these proteins was done to predict protein structures. Subsequently, the genes were cloned and expressed in a drug hypersusceptible *Escherichia coli* strain KAM32 using the vector pBR322. The recombinant clones were tested for the functionality of the efflux pump proteins by MIC determination and drug transport assays using fluorimeter.

**Results:**

The sequences of the genes were found to be around 99% identical to their counterparts in *V. cholerae*. Protein structure predicting servers TMHMM and I-TASSER depicted ten-twelve membrane helical structures for both type of pumps. Real time PCR showed that these genes were expressed in the native *V. fluvialis* isolates. In the drug transport assays, the *V. fluvialis* clinical isolates as well as recombinant *E. coli* harbouring the efflux pump genes showed the energy-dependent and sodium ion-dependent drug transport activity. KAM32 cells harbouring the recombinant plasmids showed elevated MIC to the fluoroquinolones, norfloxacin and ciprofloxacin but H-type pumps VCH and VFH from *V. cholerae* and *V. fluvialis* respectively, showed decreased MIC to aminoglycosides like gentamicin, kanamycin and streptomycin. Decrease in MIC was also observed for acriflavin, ethidium bromide, safranin and nalidixic acid.

**Significance:**

Increased resistance towards fluoroquinolones exhibited due to these efflux pumps from multidrug resistant clinical isolates of *V. fluvialis* implies that treatment procedure may become more elaborate for this simple but highly infectious disease. To the best of our knowledge, this is the first report of cloning and characterization of efflux pumps from multidrug resistant clinical isolates of *V. fluvialis*.

## Introduction

*Vibrio fluvialis* causes sporadic cases of diarrhoea clinically indistinguishable from cholera. There have been reports of increase in incidence of drug resistance in these organisms due to the presence of various factors like integrons, plasmids, SXT elements, mutations in topoisomerases and efflux pumps [1]–[4]. Some of the mechanisms by which microorganisms exhibit resistance to antimicrobials are: drug inactivation or modification, alteration of target site, alteration of metabolic pathway, reduced drug accumulation and increased active efflux of the drugs across the cell surface. Efflux pumps are one of the major determinants of the accumulation of various compounds including antibacterials inside a bacterial cell and are thought to work in synergy with other mechanisms like target gene mutations and quinolone resistance genes (*qnr*) to acquire clinical breakpoints for resistance [5], [6]. Efflux pumps were first recognized as a mode of drug resistance in early 1980s against tetracycline [7]. These pumps may be specific for one substrate or may transport a range of structurally dissimilar compounds including antibiotics of multiple classes. Efflux pumps are categorized into five major superfamilies *i.e* the major facilitator superfamily (MFS), the ATP-binding cassette family (ABC), the resistance-nodulation-division family (RND), the small multidrug resistance protein family (SMR) and multidrug and toxic compound extrusion family (MATE), the latter being the newest member of antimicrobial transporters [8], [9]. Classification of the efflux pumps is based on the sequence homology and the source of energy they utilize for drug transport. Efflux pumps usually consist of a monocomponent protein with transmembrane spanning domains. However, in gram-negative bacteria which are protected by an outer membrane, efflux transporters can be organized as tripartite systems in which the efflux pump located in the inner membrane works in conjunction with a periplasmic fusion protein and an outer membrane protein [10]. NorM, a multidrug Na^+^-antiporter, was the first MATE family pump identified from *V. parahaemolyticus* [11]. It was found to confer resistance to dyes, fluoroquinolones and aminoglycosides [11]. NorM homologues have recently been characterized in many species like *Neisseria gonorrhoeae*, *Escherichia coli* and *V. cholerae* [11]–[14]. MATE family efflux pumps utilize Na^+^/H^+^ gradient for transport and has been reported to contain three branches: the NorM branch, a branch containing several eukaryotic proteins and a branch containing *E. coli* DinF [15]. The X-ray structure of NorM from *V. cholerae* has recently been solved to the resolution of 3.63 Å [16].

Recent studies from our laboratory have suggested the role of various mobile genetic elements as well as chromosome-borne factors in multiple drug resistance of various clinical isolates of *V. fluvialis* [3], [17]. Though most of the studies of efflux pumps have been done in *V. cholerae* O1 or non-O1/non-139 strains, studies have not been envisaged in a lesser known organism like *V. fluvialis*. In the present study, D- and H- MATE-type putative transporters were cloned from clinical isolates of *V. fluvialis* and expressed in *E. coli* to deduce their functionality and examine their contribution in imparting drug resistance. Bioinformatic analysis was performed to predict their 2D and 3D structures and correlate with the findings from the functionality assays. Our results indicated that these efflux pumps were functional when expressed in a heterologous host and they displayed differential substrate specificity.

## Results

### Gene cloning, sequencing, homology and structure prediction

**Figure 1:**
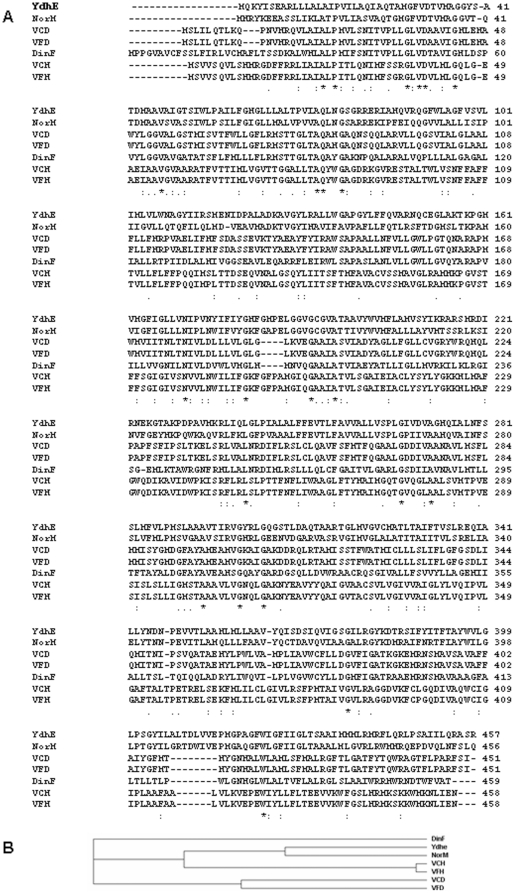
Sequence a*nalysis of* M*ATE p*um*ps fro*m vari*ous o*rganisms and expression of these genes in *parent strains*. **A.** Multiple alignment of the amino acid sequences of VCD, VFD, VCH and VFH with those of representative homologs in *Vibrio parahaemolyticus* (NorM) and *Escherichia coli* (DinF and YdhE) using CLUSTAL W2. *, identical residues. **B.** Phylogenetic analysis of the above mentioned proteins.

D- and H-MATE-type efflux pump genes were amplified from genomic DNA of *V.cholerae* N16961 and two clinical isolates L10734 and L12387 of *V.fluvialis* using published primers [18]. The sequences of recombinant clones from each of these three strains were subsequently submitted to GenBank (**EU140548–EU140550, EU263360–EU263363**). Amino acid sequence alignments of VCD (from *V. cholerae* O1) and VFD (from *V. fluvialis*) showed that they were more than 99% identical. Both these pumps showed 97–98% homology with VcmD (D-type MATE pump from non-O1/non-O139 strain of *V.cholerae*) [18]. Sequence analysis of VCH and VFH revealed that they were 99–100% identical. Also, these were almost 99% similar to previously cited VcmH efflux pump from *V.cholerae* non-O1/non-O139 [18]. In a BLAST search, all the four pumps showed 99% identity with chromosome I of *V.cholerae* N16961 indicating that these efflux pump genes could be chromosome-borne in *V. cholerae* as well as in *V. fluvialis*. CLUSTAL W alignments of protein sequences of these pumps with NorM from *V. parahaemolyticus* and YdhE and DinF family proteins of *E.coli* suggested that VCH and VFH were closer to NorM and YdhE branch (Figs. 1A and 1B). VCD and VFD seemed to have evolved differently from NorM (Fig. 1B). Analysis of nucleotide and amino acid sequences also revealed that the sequences from *V. fluvialis* were closer to *V. cholerae* O1 as compared to *V. cholerae* non-O1/non-O139.

**Figure 2:**
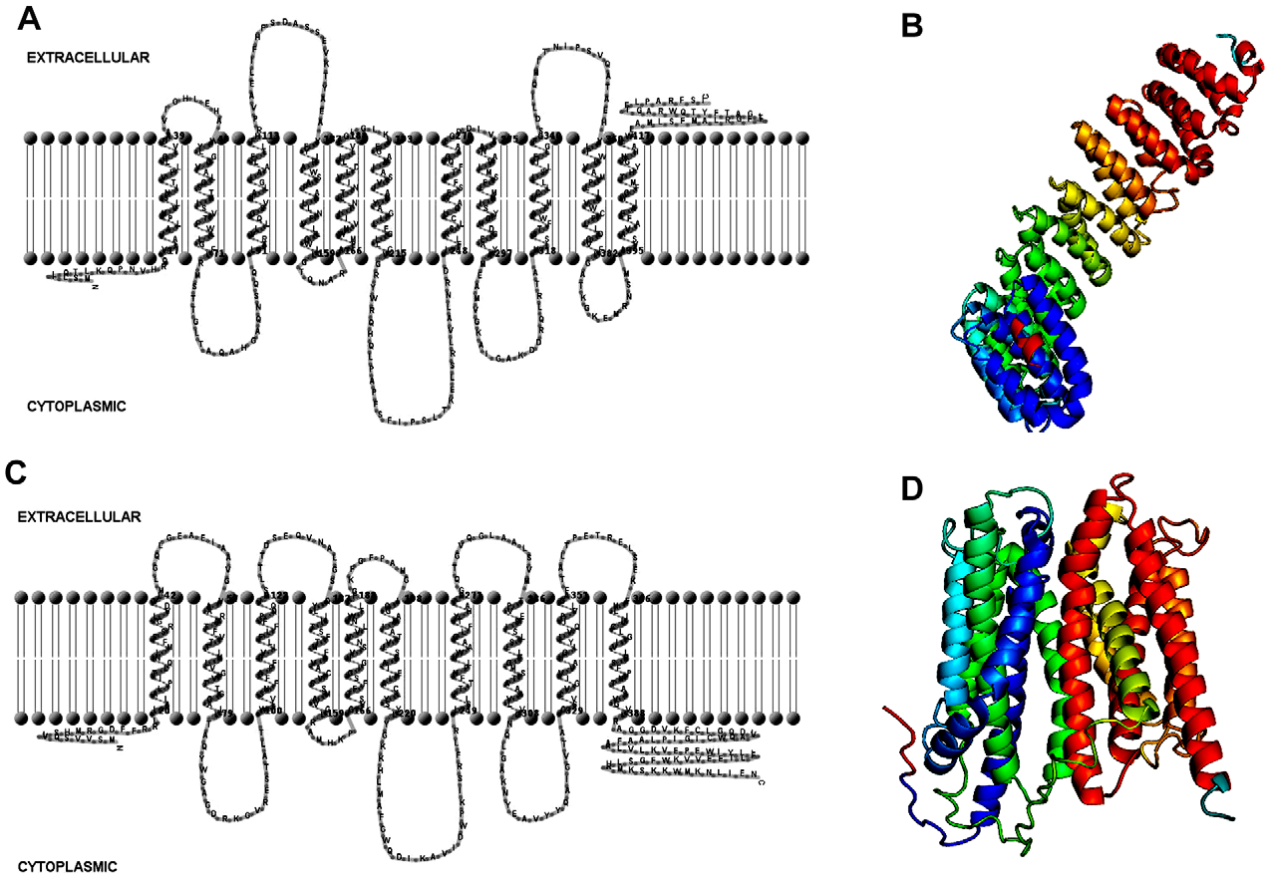
2D and 3D structure predictions of H- and D- type MATE pumps. **A and C.** Schematic representation of the predicted secondary structure of VFD and VFH respectively. The topology was predicted based on the algorithm TMHMM Server 2.0. **B and D.** Schematic representation of the predicted 3D structure of VFD and VFH respectively. The topology was predicted based on the algorithm I-TASSER.

Two-dimensional modeling for these pumps was done using TMHMM server [19]. Significant differences were observed between H- and D-type of pumps in terms of orientation of N- and C-terminii, the number of transmembrane helices and the loops in between the transmembrane helices (Figs. 2A and 2C). The VFD/VCD pumps were predicted as having eleven transmembrane helices with C-terminus located in the exterior of the cell whereas VFH/VCH pumps showed ten transmembrane helices and an internally disposed C-terminus (Figs. 2A and 2C). I-TASSER server was used for 3D protein structure prediction [20]. Results revealed that the structures of two kinds of pumps were actually different as observed in 2D predictions (Figs. 2B and 2D). Additionally, the 3D structure for VFH was similar to the structure predicted in X-ray crystallographic studies of NorM [16] whereas the structure for VFD varied from that of NorM. This observation also corroborated our earlier findings of CLUSTAL W analysis where H- and D-pumps seemed to have evolved from different branch points (Fig. 1).

### Expression of efflux pump genes in the parent *Vibrio* strain L12387

In order to confirm if the H- and D-pumps were actually expressed in the parent *Vibrio* strains, real time PCR was performed. Using *atpA* gene (encoding the α subunit of the F_1_ ATPase) as a control, mean cycle threshold (Ct) values, ΔCt and fold change (2^ΔCt^) were obtained for each gene. The results showed that expression of the *vfd* gene was 12.1-fold higher as compared to *atpA* gene and the expression of *vfh* gene was 1.1-fold higher as compared to *atpA*. This proved that both the genes *vfd* and *vfh* were expressed in the parent *V. fluvialis* strain though the levels of expression varied between the two of them.

### Change in MIC for *E.coli* host carrying recombinant proteins

MIC assays were carried out to assess the functionality of the four recombinant clones expressing VFH, VCH, VFD and VCD in an *E. coli* host KAM32. A range of changes were observed in the susceptibilities of *E. coli* cells producing either D- or H-type MATE pumps (Table 1). Elevation in MIC was observed against some compounds whereas decrease in MIC or unaltered MIC was noticed in others. With fluoroquinolones like ciprofloxacin and norfloxacin, two- to four-fold elevation in MIC was seen for both types of pumps (Table 1). Most interesting observation was decrease in MIC or increased susceptibility for nalidixic acid, acriflavin, ethidium bromide, safranin and aminoglycosides like gentamicin, kanamycin and streptomycin (Table 1). Unaltered MIC was noticed in case of chloramphenicol, neomycin and ofloxacin indicating that these drugs were not recognized by the recombinant efflux pump proteins and therefore not transported by them (Table 1). These results clearly proved that the recombinant genes were expressed in a heterologous host and were functional though the specificity of the transporters varied with the compounds. It may be pertinent to state here that the expression of these transporters appeared to be very low and resulted in only modest changes (two- to four-folds) in bacterial susceptibility to certain antimicrobials.

**Table 1:** Minimum inhibitory concentrations (MICs) for recombinant clones carrying MATE-type efflux pump genes from *V. fluvialis* and *V. cholerae*.

	MIC in µg/mL for				
Drug	pBR322	VCH	VFH	VCD	VFD
**Acriflavin**	25.0	12.5	6.25	12.5	12.5
**Chloramphenicol**	1.56	1.56	1.56	1.56	1.56
**Ciprofloxacin**	0.0045	0.009	0.009	0.009	0.009
**Ethidium Bromide**	12.5	12.5	6.25	6.25	6.25
**Gentamicin**	12.5	3.125	6.25	12.5	12.5
**Kanamycin**	25.0	12.5	12.5	25.0	25.0
**Neomycin**	25.0	25.0	25.0	25.0	25.0
**Nalidixic Acid**	3.125	0.78	1.56	0.78	1.56
**Norfloxacin**	0.019	0.078	0.039	0.039	0.039
**Ofloxacin**	0.039	0.039	0.039	0.039	0.039
**Safranin**	12.5	25.0	6.25	6.25	6.25
**Streptomycin**	12.5	6.25	6.25	12.5	12.5

VCH and VCD : pBR322 recombinant clones carrying H- and D- MATE pump genes from *V. cholerae* N16961.

VFH and VFD : pBR322 recombinant clones carrying H- and D- MATE pump genes from a clinical isolate of *V. fluvialis*.

### Presence of MATE-type efflux pumps in parent strains

**Figure 3:**
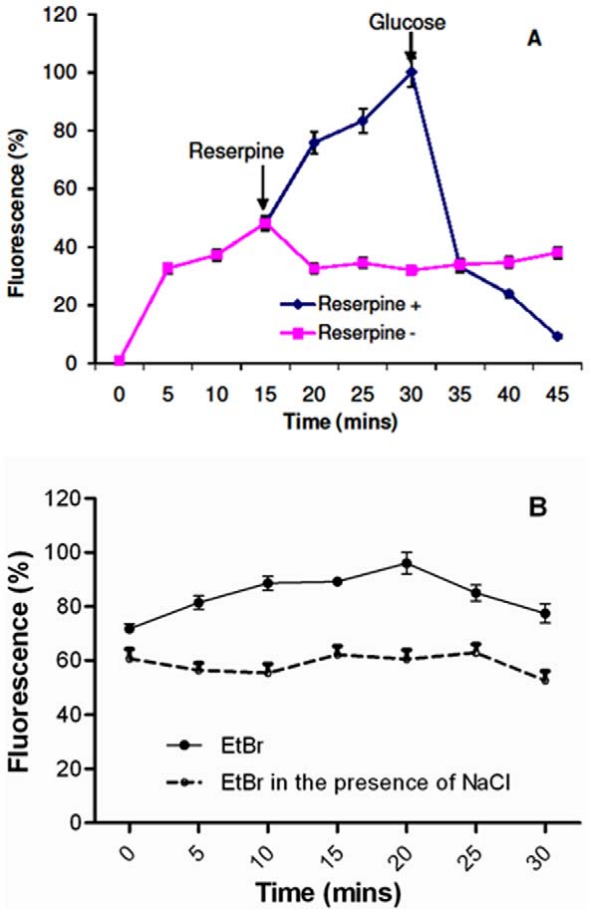
Presence of energy-dependent and Sodium-driven transporters in parent strain of *V. fluvialis*. **A.** Level of intracellular concentration of ethidium bromide in parent *Vibrio fluvialis* strain L12387. Reserpine was added as membrane decoupler leading to disruption in efflux activity and an increase in intracellular concentration of the dye. This effect was reversed on addition of glucose that energized the cells and the efflux activity resulting in a sharp decline in drug concentration inside the *Vibrio* cells. **B.** Decrease in the levels of intracellular concentration of ethidium bromide in the parent strain in the presence of 100 mM NaCl. Shown are the levels of accumulated dye in the presence and the absence of the salt.

To ascertain the presence of MATE efflux pumps in the parent strains, ethidium bromide accumulation assays were carried out as described in Materials and Methods. Both the parent strains L10734 and L12387 were used for the assay yielding the same results. Hence the data has been presented only for the strain L12387. MATE pumps utilize energy from the proton motive force (PMF) for transport of different substrates [12]. Altering the generation of PMF using reserpine blocks pump activity resulting in the enhanced accumulation of substrates [9], [17]. Same effect was observed when the *V. fluvialis* cells were treated with reserpine that aborted the PMF and increased the ethidium bromide concentration inside the cells (Fig. 3A). When reserpine was removed and glucose was added to energise the cells, the efflux activity of the pumps in the parent strains was regained and the active drug efflux was initiated that resulted in a drastic drop in the intracellular concentration of the dye (Fig. 3A). Also, the efflux activity was enhanced on addition of 100 mM NaCl (Fig. 3B). Therefore, efflux of the dye was found to be energy-driven (Fig. 3A) and Na^+^-dependent (Fig. 3B).

### Reduction of intracellular drug concentration in the recombinant *E. coli*

**Figure 4:**
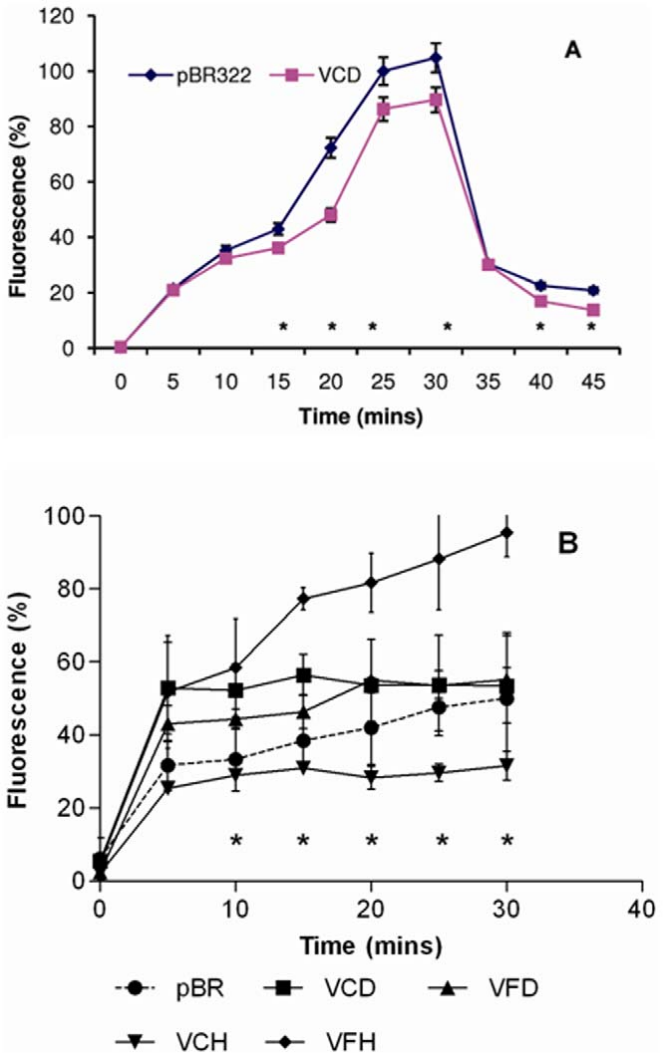
Involvement of recombinant efflux pumps in the transport of various compounds. **A.** VCD-induced efflux of norfloxacin from recombinant *E. coli* harbouring *vcd* gene. pBR322: Accumulation of norfloxacin in KAM32 cells transformed with *pBR322* empty vector, VCD: Accumulation of norfloxacin in KAM32 cells transformed with *pBR322-vcd*. *, the values of fluorescence for VCD were significantly different for as compared to pBR322 control (*P*<0.05). **B.** Accumulation of ethidium bromide in cells transformed with *pBR322* empty vector (pBR), *pBR322-vcd* (VCD), *pBR322-vfd* (VFD), *pBR322-vch* (VCH) and *pBR322-vfh* (VFH) *, the values of fluorescence for VFH were significantly different as compared to pBR322 control (*P*<0.05). For other recombinants it was significantly different at either of indicated time points (*P*<0.05).

The observed drug specificity of recombinant clones of VCD, VFD, VCH and VFH for fluoroquinolones in MIC assays was consistent with the drug recognition profiles of other members of the MATE family [12], [14], [18]. To confirm the drug susceptibility testing results, the levels of accumulation of norfloxacin were measured in KAM32 cells that carried recombinant pBR322 encoding VCD, VFD, VCH and VFH. As shown for VCD, the levels of norfloxacin accumulation increased radically in the recombinant cells after the addition of reserpine (Fig. 4A). There was a drop in the levels of norfloxacin accumulation on addition of glucose (Fig. 4A). Interesting observation was made with ethidium bromide accumulation in recombinant KAM32 cells compared to the *pBR322* control. MIC results had shown no change in MIC in VCH and decrease in MIC in case of VFH, VCD and VFD which was also reflected in the accumulation assay where VFH, VCD and VFD exhibited more accumulation of the dye as compared to VCH and control *pBR322* (Fig. 4B).

### D- and H-type efflux pumps are sodium-dependent transporters

**Figure 5:**
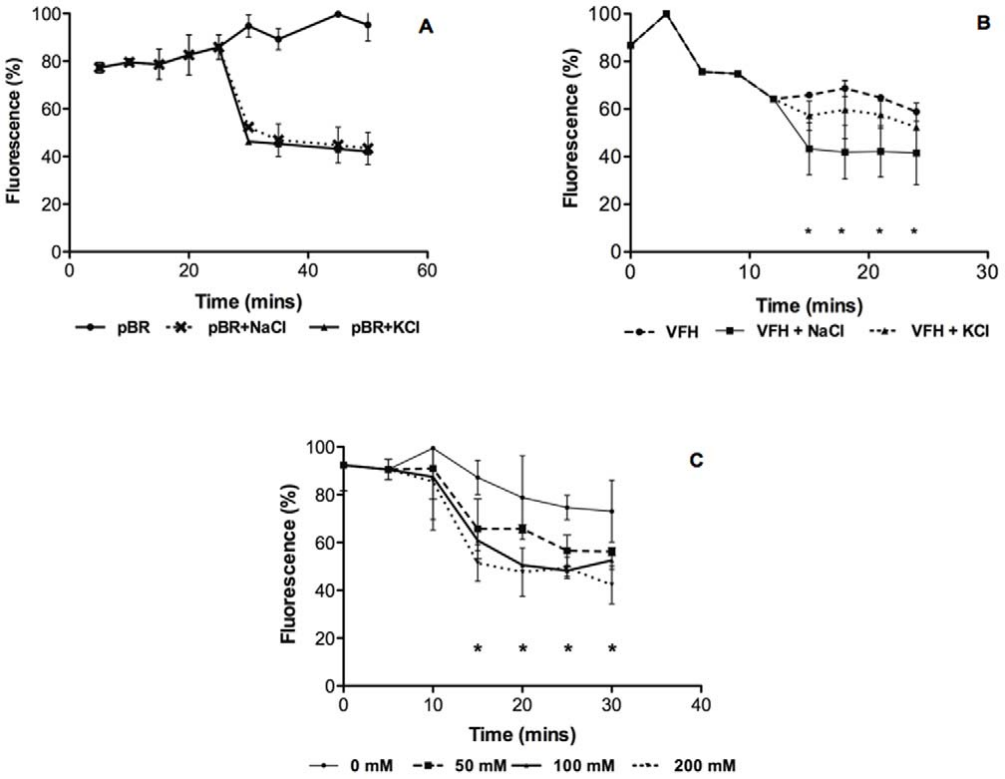
Sodium-ion dependence of recombinant efflux pumps Induction of norfloxacin efflux in the presence of 100 mM sodium chloride (+NaCl) or 100 mM potassium chloride (+KCl) in the *E. coli* KAM32 cells transformed with **A.** *pBR322* or **B.** *pBR322-vfh*. **C.** Effect of increase in Sodium ion concentration on the efflux pump activity of VFH. *, The activity of VFH in the presence of 100 mM and 200 mM NaCl was significantly different from that of the control without NaCl (*P*<0.05).

It has been shown in case of *V. cholerae* non-O1/non-O139 that D- and H-type efflux pumps are Na^+^/drug antiporters [18] but there is paucity of information on their involvement in imparting drug resistance in *V. fluvialis*. Thus, the levels of accumulation of norfloxacin in KAM32 were investigated in the presence of Na^+^ or K^+^ ions. The addition of 100 mM NaCl to KAM32 cells harboring D- and H-type pump encoding genes resulted in a greater efflux of the drug from these cells compared to the levels of efflux observed in the presence of KCl or without salt (Fig. 5B). This effect was not observed in the control *pBR322*/KAM32 cells where same accumulation was observed in the presence of NaCl or KCl (Fig. 5A). Taken together, the data suggest that these efflux pump proteins are Na^+^-dependent transporters. Furthermore, to test the hypothesis that efflux of an antimicrobial by these proteins is Na^+^ -dependent, cells were first loaded with 100 µM norfloxacin using reserpine as described in Materials and Methods. Thereafter, different concentrations of NaCl (50 to 200 mM) were added to test their effects on drug efflux. The results showed that the efflux activity increased with the increase in NaCl concentration (Fig. 5C). Thus, drug accumulation and efflux assays confirmed that D- and H-type efflux pumps of *V.cholerae* and *V.fluvialis* were indeed Na^+^/drug antiporters.

## Discussion

The present study was aimed at detailed characterization of the efflux pump proteins from the not-so-well studied pathogen *Vibrio fluvialis* responsible for sporadic cases of diarrhoea and also known to be associated with pediatric diarrhoea [1]–[3]. Though a few studies have been reported for the MATE pumps from *V. cholerae* O1 and non-O1/non-O139 strains, no information is available on the cloning and characterization of recombinant efflux pumps from *V. fluvialis* to understand their role in multiple drug resistance displayed by these organisms. To achieve this information, two types of MATE pumps were isolated from multidrug resistant clinical isolates of *V. fluvialis* with the objective of studying their structure and function. Protein sequence analysis of VCD, VFD, VCH and VFH along with other known homologs from *V. parahaemolyticus* and *E. coli* suggested that VCH and VFH belonged to common ancestral origin as that of NorM, a very well studied MATE pump from various pathogens including *Vibrios* [11]–[16]. VFD and VCD seemed to have evolved differently. Asp32, Glu251 and Asp367 from *V.parahaemolyticus* NorM have been implicated in sodium-dependent transport of antimicrobials and hence have a crucial role in imparting multidrug resistance [15]. Protein sequence alignment revealed that Asp32 was present in all the four pumps. Glu251 of NorM was replaced by leucine in VCD, VFD and DinF and by tryptophan in VCH and VFH. Asp367 of NorM was replaced with alanine in VCD and VFD, with glycine in DinF and with serine in VCH and VFH.

The crystal structure and previous predictions pertaining to its secondary structure have suggested a twelve transmembrane helical structure for NorM [14], [16]. Models of the secondary structure of VFD and VFH revealed that these were not 12-helical structures like NorM. 3-Dimensional structure of VFD and VFH corroborated the findings of phylogenetic analysis that predicted structural similarity of VFH and structural diversity of VFD with respect to NorM. This proved our hypothesis that D-and H-MATE-type of efflux pumps have evolved differently. This was also confirmed later in the functionality studies of these proteins as assessed by MIC and drug transport assays.

Previous reports about NorM from *V. parahaemolyticus* have depicted that it confers resistance to hydrophilic fluoroquinolones such as norfloxacin and ciprofloxacin, but not to hydrophobic quinolones such as sparfloxacin and nalidixic acid [11]. Though NorM is the most well studied MATE pump in terms of its structure and function, other MATE-type efflux pumps (VcmH, VcmD, VcrM, VcmA, VcmB and VcmN) have also been characterized from non-O1/non-O139 *V.cholerae* [18]. Our study with H- and D-pumps from *V. fluvialis* also revealed that these putative efflux pumps showed two- to four-fold increase in MIC/increased resistance towards norfloxacin and ciprofloxacin and increased susceptibility towards nalidixic acid. Interestingly, only VCH and VFH showed increased susceptibility towards aminoglycosides suggesting increased intake of these compounds only by H- and not D-type pumps. This kind of effect for increased kanamycin permeability has also been observed earlier [15]. Decrease in MIC can be explained in terms of these drugs not being recognised by the pumps as their substrates but being permeabilised by some porin. As the bacterial cell is exposed to an antibiotic, there are complex regulatory mechanisms that come into play. These involve the MAR operon that regulates not only efflux pumps, but also the porins. We hypothesise that the decrease in MIC may be an outcome of such a mechanism where in response to aminoglycosides, some porins get activated/overexpressed and allow the influx of these compounds thus increasing the intracellular concentration of these drugs [21]. Previous studies had revealed that Asp32 was responsible for norfloxacin resistance as mutants of Asp32 showed low levels of norfloxacin resistance [15]. As all these four pumps from *V. fluvialis* carried Asp at position 32, they exhibited two- to four-fold rise in norfloxacin resistance. These pumps exhibited increased susceptibility towards dyes like ethidium bromide, acriflavin and safranin again suggesting a channel-like function as noticed in the case of aminoglycosides. Therefore, these interesting observations regarding the different structures for two MATE pumps resulting in differential substrate specificity need to be investigated further to probe their channel-like function. In addition, it could be interesting to look for efflux pump inhibitors to overcome the drug resistance problem.

## Materials and Methods

### Bacterial strains and plasmids

*Vibrio fluvialis* isolates were obtained from patients with acute cholera-like diarrhoea admitted to Infectious Diseases Hospital, Kolkata, India, in 2006. All the isolates were kindly provided by Dr. T. Ramamurthy, National Institute of Cholera and Enteric Diseases, Kolkata, India, in the form of bacterial stabs. *V. cholerae* O1 El Tor strain N16961 and *V. fluvialis* isolates L10734 (resistant to neomycin, co-trimoxazole, nalidixic acid, trimethoprim and partially resistant to ampicillin, kanamycin and ciprofloxacin) and L12387 (resistant to neomycin, co-trimoxazole, nalidixic acid, trimethoprim, ampicillin, streptomycin and sulfisoxazole and partially resistant to kanamycin, chloramphenicol, norfloxacin and ciprofloxacin) were used as the source of genomic DNA. *Escherichia coli* JM109 was used for electroporation experiments and *E. coli* strain KAM32 (Δ*acrB*, Δ*ydhE*, *hsd* ^−^) was used for gene expression [22]. Vector pDrive (QIAGEN GmbH, Hilden, Germany) was used for TA cloning of PCR products and their sequencing. Plasmid pBR322 was used for expression of the efflux pump genes and functional characterization of the recombinant proteins. *V. cholerae* and *E. coli* cells were grown in Luria-Bertani medium at 37°C under aerobic condition. The study was approved by the Institutional Biosafety Committee (IBSC) and Review Committee on Genetic Manipulation (RCGM) governed by guidelines laid down by the Department of Biotechnology, Govt. of India.

### DNA Preparation

Genomic and plasmid DNA were prepared from *V.cholerae* and *V.fluvialis* cells as described earlier [23]. Plasmid Mini kit or Maxi Kit (QIAGEN) were used for DNA purifications according to manufacturer's instructions. Gel extraction was done according to manufacturer's protocol (QIAGEN).

### Polymerase chain reaction (PCR)

PCR assays were carried out using specific primers for D- and H-type MATE pumps, and genomic DNA templates as described earlier [18]. Each PCR involved an initial denaturation at 95°C for 4 min, followed by 25 amplification cycles each consisting of an initial denaturation at 95°C for 30 sec, annealing at 50°C for 1 min and extension at 72°C for 1 min 30 sec. Final polymerisation was carried out at 72°C for 10 min. PCR reactions were performed using PTC-225 DNA engine Tetrad™ (MJ Research Inc., Waltham, MA). Recombinant Taq polymerase (Fermentas International Inc., Burlington, Ontario, Canada) was used along with the buffer containing ammonium sulfate, and magnesium chloride was added at a final concentration of 2 mM.

### Real Time PCR

RNA was isolated from the *V. fluvialis* isolate L12387 using Trizol method. cDNA was generated using RevertAid H Minus first strand cDNA synthesis kit (MBI, Fermentas). The cDNA was amplified with TAQXpedite real time PCR master mix (Epicentre Biotechnologies, Madison, WI), using the primers already described [18]. *atpA* was used as a positive control.

### Gene cloning

The PCR products of the right size (1573 bp for D amplicons and 1531 for H amplicons) were purified from low melting point agarose gel using the gel extraction kit (QIAGEN). The amplicons were cloned in pDrive vector, electroporated in *E. coli* JM109 cells and plated on LB containing ampicillin (100 µg/mL). The recombinants were selected using blue/white screening and restriction enzymes specific for each gene. Two positive clones from each type of recombinant were sequenced. The sequenced clones of D- and H-type efflux pump genes were designated *vcd*, *vfd*, *vch* and *vfh*, where c stands for *V. cholerae* and *f* stands for *V. fluvialis*. For construction of expression clones, DNA from the recombinants described above were digested with *Bam*HI and approx. 1.4 kb insert was ligated into *Bam*HI-digested pBR322 dephosphorylated with calf alkaline phosphatase (Fermentas). The ligated product was electroporated in *E.coli* JM109 and plated on LB plates containing ampicillin (50 µg/mL).

### Drug susceptibility assays

The MICs of these recombinants were determined by two-fold dilution method against various drug concentrations. The highest concentration of each drug for the assay was prepared by addition of 1000X concentrated stock of the drug to the appropriate volume of LB medium containing ampicillin (50 µg/mL). Rest of the concentrations for that drug were made by serial double dilution method using LB medium containing ampicillin (50 µg/mL). The recombinants were transformed in *E. coli* KAM32 cells and plated on LB plates containing ampicillin (50 µg/mL). The transformants were grown in LB medium containing ampicillin (50 µg/mL) to an OD_600_ of 0.008–0.013. 2.0 mL of each drug dilution was distributed in 24 well sterile tissue culture plates (Corning, NY). 50 µL of bacterial culture was then added to each drug concentration and the bacterial growth was monitored after 16–18 hours. The MICs were determined as the lowest concentration of the drug that inhibited bacterial growth. The assays were repeated atleast three times.

### Ethidium Bromide accumulation assay in parent strains

Assays for ethidium bromide accumulation were performed as described before with minor modifications [12]. *V.cholerae* N16961 and two *V.fluvialis* strains L10734 and L12387 were taken for this study. Cells were grown in LB broth at 37°C to an OD_600_ of 1.0, harvested, washed thrice with buffer containing 0.1 M Tris.HCl, pH 7.0, and suspended in the same buffer to an OD_600_ of 1.0. A zero minute sample served as a negative control. Ethidium Bromide was then added at a final concentration of 20 µg/mL. Samples of 1 mL were collected at 5 min intervals throughout the assay and these aliquots were stored in prechilled microcentrifuge tubes. After 15 min, reserpine was added to a final concentration of 20 µg/mL to disrupt the proton gradient across the membrane. Fifteen minutes later, reserpine was removed by centrifugation at 10,000 rpm for 4 min and the cells were resuspended in 0.1 M Tris.HCl, pH 7.0 buffer containing 0.4% glucose and samples were collected every 5 min. Samples of each time point were then centrifuged at 10,000 rpm for 5 min at 4°C, washed once with 0.1 M Tris.HCl, pH 7.0 buffer and resuspended in 1 mL of 100 mM glycine.HCl, pH 3.0. These samples were then shaken vigorously for 2 h at 37°C to release the fluorescent content followed by centrifugation at 15,000 rpm for 10 min at room temperature. The fluorescence of the supernatants was measured at excitation and emission wavelengths of 500 nm and 580 nm, respectively, using a RF-5301 PC spectrofluorometer (Shimadzu, Singapore). The amount of maximum fluorescence was normalized to 100%.

### Drug accumulation assays with recombinant clones

For the assays with recombinants, *E. coli* KAM32 cells carrying *pBRVCD*, *pBRVFD*, *pBRVCH*, *pBRVFH* or *pBR322* were each grown in LB medium containing ampicillin (50 µg/mL) to an OD_600_ of 1.0 and processed exactly in the same way as described above except that 100 µM norfloxacin was used instead of ethidium bromide.

### Accumulation of ethidium bromide in recombinant clones

*E. coli* KAM32 cells containing *pBRVCD*, *pBRVFD*, *pBRVCH*, *pBRVFH* or *pBR322* were grown in LB broth at 37°C to an OD_600_ of 1.0, harvested by centrifugation, washed thrice with buffer containing 0.1M Tris.HCl, pH 7.0, and suspended in the same buffer to an OD_600_ of 1.0. Ethidium bromide (final concentration 20 µg/ml) was added to the cell suspension and incubation was carried out for 35–45 min to study the accumulation pattern. Collection of the samples and measurement of the fluorescence intensity was done exactly as described above.

### Efflux of norfloxacin in the presence of Na^+^

For the determination of norfloxacin efflux, recombinant KAM32 cells were grown, harvested and washed as described before. The cells were incubated in 0.1M Tris.HCl, pH 7.0 buffer supplemented with 100 µM norfloxacin and 20 µg/mL reserpine at 37°C for 30 min to load the cells with norfloxacin. The cells were then pelleted, washed twice, and resuspended in the Tris buffer to an OD_600_ of 2.0. After 15 min, the cell suspension was divided into three equal portions; two portions received either NaCl or KCl at final concentrations of 100 mM whereas the third portion received equal volume of water. Samples of 1 ml were taken at 3 mins intervals, centrifuged at 10,000 rpm for 5 mins at 4°C, and washed once with the same buffer. The fluorescence of the supernatants were measured at an excitation and an emission wavelengths of 277 nm and 448 nm, respectively.
